# Genetic dissection of mutagenic repair and T-DNA capture at CRISPR-induced DNA breaks in *Arabidopsis thaliana*

**DOI:** 10.1093/pnasnexus/pgae094

**Published:** 2024-02-26

**Authors:** Lycka Kamoen, Lejon E M Kralemann, Robin van Schendel, Niels van Tol, Paul J J Hooykaas, Sylvia de Pater, Marcel Tijsterman

**Affiliations:** Department of Plant Sciences, Institute of Biology Leiden, Leiden University, Leiden 2333 BE, The Netherlands; Department of Plant Sciences, Institute of Biology Leiden, Leiden University, Leiden 2333 BE, The Netherlands; Department of Human Genetics, Leiden University Medical Center, Leiden 2300 RC, The Netherlands; Department of Human Genetics, Leiden University Medical Center, Leiden 2300 RC, The Netherlands; Department of Plant Sciences, Institute of Biology Leiden, Leiden University, Leiden 2333 BE, The Netherlands; Department of Human Genetics, Leiden University Medical Center, Leiden 2300 RC, The Netherlands; Department of Plant Sciences, Institute of Biology Leiden, Leiden University, Leiden 2333 BE, The Netherlands; Department of Plant Sciences, Institute of Biology Leiden, Leiden University, Leiden 2333 BE, The Netherlands; Department of Plant Sciences, Institute of Biology Leiden, Leiden University, Leiden 2333 BE, The Netherlands; Department of Human Genetics, Leiden University Medical Center, Leiden 2300 RC, The Netherlands

**Keywords:** T-DNA, *Arabidopsis thaliana*, classical nonhomologous end joining (cNHEJ), polymerase theta-mediated end joining (TMEJ), DNA repair

## Abstract

A practical and powerful approach for genome editing in plants is delivery of CRISPR reagents via *Agrobacterium tumefaciens* transformation. The double-strand break (DSB)-inducing enzyme is expressed from a transferred segment of bacterial DNA, the T-DNA, which upon transformation integrates at random locations into the host genome or is captured at the self-inflicted DSB site. To develop efficient strategies for precise genome editing, it is thus important to define the mechanisms that repair CRISPR-induced DSBs, as well as those that govern random and targeted integration of T-DNA. In this study, we present a detailed and comprehensive genetic analysis of Cas9-induced DSB repair and T-DNA capture in the model plant *Arabidopsis thaliana*. We found that classical nonhomologous end joining (cNHEJ) and polymerase theta-mediated end joining (TMEJ) are both, and in part redundantly, acting on CRISPR-induced DSBs to produce very different mutational outcomes. We used newly developed CISGUIDE technology to establish that 8% of mutant alleles have captured T-DNA at the induced break site. In addition, we find T-DNA shards within genomic DSB repair sites indicative of frequent temporary interactions during TMEJ. Analysis of thousands of plant genome–T-DNA junctions, followed up by genetic dissection, further reveals that TMEJ is responsible for attaching the 3′ end of T-DNA to a CRISPR-induced DSB, while the 5′ end can be attached via TMEJ as well as cNHEJ. By identifying the mechanisms that act to connect recombinogenic ends of DNA molecules at chromosomal breaks, and quantifying their contributions, our study supports the development of tailor-made strategies toward predictable engineering of crop plants.

**Significance Statement**

In order to produce safe crop plants by CRISPR-mediated genome engineering, detailed knowledge is needed on the mechanisms acting to repair DNA double-strand breaks in plant genomes. In addition, it is desirable to understand how and with which frequently ectopically provided DNA, which can encode the CRISPR reagents, can integrate at an inflicted DNA break site (or elsewhere in the genome). This study identifies the mechanisms that act on CRISPR-induced DNA breaks in the model plant *Arabidopsis thaliana* to either produce genome edits or capture transfer DNA at a genomic break site. Detailed, quantitative analysis of the mutational outcomes sheds light on the biochemistry of genome engineering which may benefit development of safe crops.

## Introduction

*Agrobacterium tumefaciens*–mediated transformation (AMT) provides a convenient and efficient way to deliver foreign DNA into plant cells. Within *Agrobacterium*, the transfer DNA (T-DNA) is located on a plasmid confined by so-called left and right border (LB and RB) repeat sequences. The virulence proteins VirD1 and VirD2 generate ssDNA nicks at these border sequences to liberate T-DNA as a single-stranded DNA molecule (1, 2). VirD2 remains covalently attached to the 5′ (RB) end of the T-DNA and pilots it to the plant nucleus (3), where it is converted to dsDNA (4, 5) enabling transient expression. Stable transformation is accomplished when T-DNA integrates into the plant genome at randomly occurring double-strand breaks (DSBs) (6).

DSB repair is often described as having two conceptually distinct branches: one that uses a homologous template for repair (i.e. homologous recombination) and one that directly joins the ends independently of a repair template (i.e. end joining [EJ]). The best studied EJ pathway is classical nonhomologous end joining (cNHEJ), in which binding of the Ku70/80 heterodimer to the DNA ends prevents DNA end resection and enables recruitment of necessary factors for repair, including DNA ligase IV (Lig4). Lig4 can ligate blunt and compatible ends, thus restoring the original sequence. Incompatible ends are subjected to minor processing prior to ligation, resulting in small deletions or insertions (7–9). Another EJ pathway has recently become the subject of intense investigation after the identification of the presently only known pathway-exclusive component: DNA polymerase theta (Polθ). Many of the previously genetically ill-defined “alternative EJ” (Alt-EJ) activities proved to be reliant on Polθ functionality, and it has also become clear that for certain substrates polymerase theta-mediated end joining (TMEJ) is the only pathway capable of restoring an intact DNA molecule. In the model plant *Arabidopsis thaliana*, Polθ is encoded by the *TEBICHI* gene (10). TMEJ makes use of minute stretches of sequence homology (so-called microhomology) in the flanks of 5′- to 3′-resected DNA to anneal and subsequently prime DNA synthesis creating a stable interaction between DNA ends (11–14). Because of this intrinsically mutagenic mode of action, TMEJ produces a typical mutation profile: next to deletions that are characterized by the presence of microhomology at the junctions, deletions embedding a (templated) insertion (delins) are also found, as a result of extension, dissociation and reannealing of the two ends at a new position (15–18).

It was recently shown that TMEJ is responsible for T-DNA integration in *A. thaliana* (19), while variable results have been reported on whether or not cNHEJ is involved (20–27). T-DNA integrates at random positions in the genome, frequently in complex multimeric configurations, and is also often accompanied by translocations (28–30) making T-DNA a suboptimal and even potential problematic vehicle for establishing ectopic expression. Targeted T-DNA integration in a predefined locus, abolishing such positional variations, would therefore provide a major advantage for crop engineering. Since early studies demonstrated targeted T-DNA integration mediated by DSB induction (4, 6, 31–33), the introduction of the very efficient CRISPR/Cas9-system alongside T-DNA transformation provides a reasonable next step forward to clean and precise integration of exogenously provided DNA. At present, little is known about DSB repair of CRISPR/Cas9-induced breaks in higher plants, nor is there knowledge about how genomic repair relates to T-DNA integration at these breaks. Therefore, we performed high-resolution next generation sequencing (NGS)-based mutational analysis on multiple Cas9 targets in a variety of repair proficient and deficient conditions to present a detailed picture of CRISPR/Cas9-induced DSB repair and T-DNA capture in *A. thaliana*.

## Results

### Mutagenic repair of CRISPR-induced DSBs in root cells

**Fig. 1.**
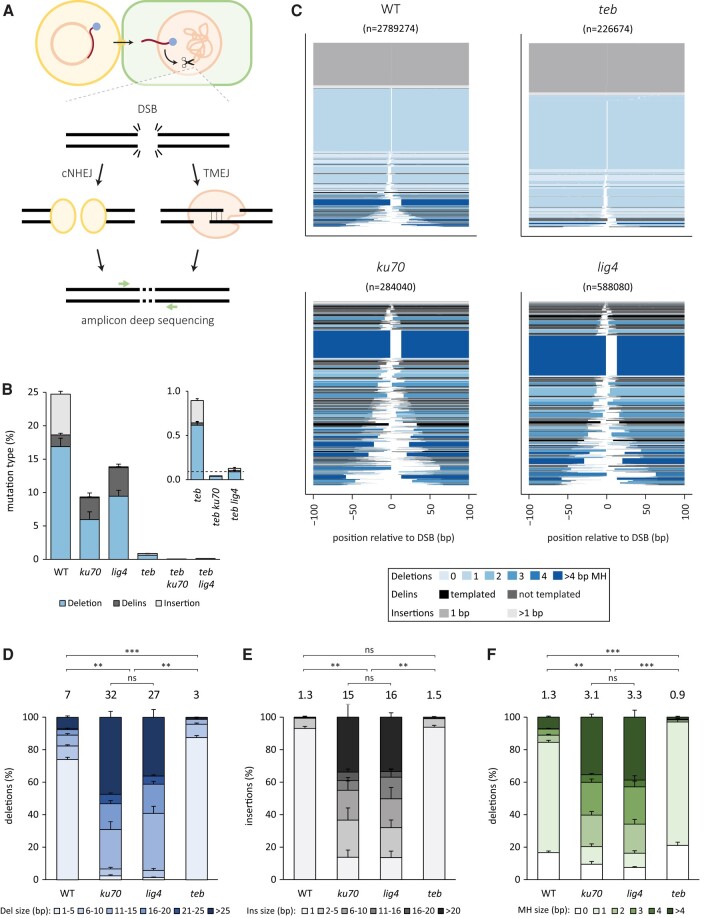
Mutational footprints in the PPO locus. A) A schematic overview of the experiment. T-DNA is transferred from *A. tumefaciens* to roots of *A. thaliana* seedlings. Cas9 and sgRNA is expressed from the T-DNA and a DSB is induced in the PPO locus, which can be repaired by one of the EJ pathways. After isolation of the genomic DNA, the region around the DSB is amplified and deep sequenced. B) Percentage and type of mutations. The error bars represent the SE between biological replicates. The dashed line represents control data without DSB induction. C) Mutational spectra at the PPO locus for the indicated genotypes combined for all biological replicates. The relative position on the *x*-axis includes the expected DSB position at 0 bp. All mutational events are stacked and sorted based on their size. The number of sequencing reads representing a specific outcome is represented by the thickness of the respective bar. The events are color-coded based on the type of event and the extent of microhomology (MH). D) Histogram depicting the deletion size class distribution of all deletion events. E) Histogram depicting the insertion size of all insertion events. F) Histogram depicting the microhomology use of all deletion events. D–F) The error bars represent the SE between biological replicates. The weighted average is indicated on top of the bars. Statistical significance between the weighted averages was calculated by Kruskal–Wallis test, followed by a post hoc Wilcoxon rank-sum test with Bonferroni correction for multiple testing. ns, not significant. **P ≤ 0.01, ***P ≤ 0.001.

To study DSB repair in *A. thaliana*, we transformed root tissue of wild-type (Col-0) plants and various EJ mutants with a T-DNA encoding *Streptococcus pyogenes* Cas9, a single-guide RNA (sgRNA) targeting the protoporphyrinogen oxidase (*PPO*) gene, and the *bar* gene product conferring phosphinothricin (PPT) resistance. Transformed callus tissue was harvested and pooled after 3 weeks, followed by amplicon deep sequencing of the target locus (see Fig. 1A for a schematic representation). For wild-type, we found a mutation frequency of 25% at the target locus (Fig. 1B); of note, the presence of unaffected PPO alleles can be due to incomplete cleavage by Cas9, error-free DSB repair, or the presence of DNA from residual untransformed root tissue. The percentage of altered alleles is decreased in roots deficient for cNHEJ factors *ku70* and *lig4*: respectively, 9 and 14% of the reads contain mutations, highlighting the contribution of mutagenic cNHEJ in wild-type cells. The contribution of TMEJ to mutation induction is more difficult to establish due to reduced DSB formation in TMEJ-deficient *teb* mutant plants: as we will reveal later, efficient DSB induction is dependent on stable expression of Cas9, which in turn is dependent on *TEB*-mediated T-DNA integration. Nevertheless, nonintegrated transient expression—which can still be observed in *teb* plants (19)—of Cas9 leads to 1% mutated alleles in *teb* mutant roots, and we find these to be almost entirely the result of cNHEJ, as the number of mutations in double mutants is further reduced to 0.03 and 0.12% for *teb ku70* and *teb lig4*, respectively, which is in the same order of magnitude as the control without DSB induction (0.09%; dashed line; Fig. 1B; inset). This result also means that TMEJ and cNHEJ together are responsible for the vast majority of mutations observed in wild-type plant cells. This conclusion is fully supported by mutation profile analysis, as we find the mutation spectrum obtained from wild-type to be a combination of the two characteristic mutation profiles that are representative for these two different EJ pathways (Fig. 1C): cNHEJ, the active EJ pathway in *teb* mutants, producing small deletions (Fig. 1C and D) and 1 bp insertions (Fig. 1C and E) at CRISPR-induced DSBs, and on the other hand, TMEJ, responsible for the mutations in *ku70* and *lig4* backgrounds, producing more sizable deletions (Fig. 1C and D) that are typified by microhomology (Fig. 1C and F) and regularly contain insertions, many of which are recognizable as templated insertions (Fig. 1C).

We next addressed the potential influence of sequence context in mutagenic DSB repair, for which we targeted three additional genomic loci: *alcohol dehydrogenase 1* (*ADH1*), *cruciferin 3* (*CRU3*), and *glabra 2* (*GL2*). The obtained mutation spectra were highly similar in all genotypes examined, indicating a minor impact of the location of the break (Fig. S1). Notable exceptions were the fractions of cNHEJ-mediated 1 bp insertions and small deletions, and the degree of microhomology found at the deletion junctions (Figs. S1 and S2A–L). The latter is not unexpected, since the availability of microhomologies is dictated by the sequence itself (34). Also for these targets, cNHEJ (inspected in the *teb* mutant background) preferentially produces 1–5-bp-sized deletions with 0–1 bp of microhomology, whereas TMEJ (examined in *ku70* mutant cells) results in more sizeable deletions characterized by microhomology at the junctions and the regular presence of templated insertions (most preferential outcomes are shown in Fig. S1B). cNHEJ also produces insertions but the vast majority of these consist of a single nucleotide identical to the one flanking it (Fig. S1B), which is in perfect agreement with the fill-in of a staggered Cas9-induced break as was previously suggested (35). Based on the four target sites together, we conclude that ∼70% of CRISPR-induced mutations in *Arabidopsis* root cells are brought about by cNHEJ and 30% by TMEJ (Fig. S2M).

### Mutagenic repair of CRISPR-induced DSBs in germ cells

**Fig. 2.**
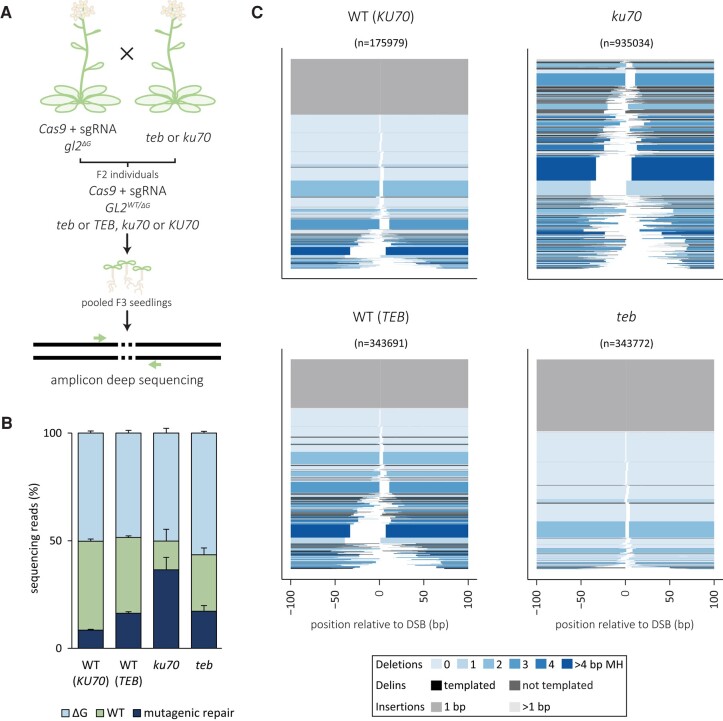
Mutational footprints in the GL2 locus in stable Cas9-expressor lines. A) A schematic overview of the experiment. Homozygous Cas9-expressor lines containing a sgRNA for the GL2 locus and a single nucleotide deletion in the GL2 locus are crossed with homozygous *teb* or *ku70* mutant lines. For each EJ mutant or wild-type (*teb*, TEB, *ku70* and KU70), three F_2_ individuals were selected that were homozygous for Cas9 and sgRNA, heterozygous for GL2, and either homozygous or wild-type for the respective EJ gene. Four hundred F_3_ seedlings of each F_2_ plant were pooled, and of the isolated DNA, the GL2 locus was amplified and deep sequenced. B) Percentage of sequencing reads of the GL2 locus for Cas9-expressor lines with the indicated EJ deficiency or proficiency. The parental lines were heterozygous for the GL2 allele leading to ∼50% of reads with a single nucleotide deletion. The percentage of reads containing a mutational footprint different from the parental lines is indicated in dark blue. The error bars represent the SE between biological replicates. C) Spectra of mutations occurring in the GL2 locus for the indicated genotypes combined for all biological replicates. The relative position on the *x*-axis includes the expected DSB position at 0 bp. All mutational events are stacked and sorted based on their deletion size. The number of reads representing a specific outcome is represented by the thickness of the respective bar. The events are color-coded based on the type of event and the extent of microhomology (MH).

We next validated our results using a different experimental setup, in which we studied the repair of CRISPR-induced DSBs in plants that express Cas9 under the control of the *DD45* promoter, as well as an sgRNA that targets the *GL2* locus at the exact same sequence as in the aforedescribed root transformations. Apart from expanding the analysis to other cell types—the *DD45* promoter drives expression in germ cells, gametes, and the early embryo—this setup also controls for a concern regarding the transformation experiments, i.e. different expression levels of T-DNA encoded Cas9 in genotypes in which T-DNA integration itself may be perturbed (19–26). To this end, we generated plants by crossing, which (i) contained the Cas9 and sgRNA expression cassette (described in Refs. (36, 37)), (ii) had one wild-type (*GL2*^+^) and one mutant (targeting-resistant) *GL2* allele (*GL2*^ΔG^), and (iii) were either proficient or deficient for either cNHEJ or TMEJ (Fig. 2A). Subsequently, we performed deep sequencing of *GL2* amplicons generated from pools of 400 F_3_ progeny. For repair-proficient plants, we find 17 to 32% of targeting-susceptible alleles to be mutated (Fig. 2B)—due to heterozygosity of *GL2* in F_2_ lines (*GL2*^+/ΔG^), ∼50% of the reads contain a 1-bp deletion (ΔG). The spectrum of mutations is very similar to that derived by root transformation, indicating that the mechanisms acting to repair CRISPR-induced DSBs are very similarly active in these different tissues. Indeed, genetic inactivation of either cNHEJ or TMEJ affected the mutation profile in opposite directions, in a similar way to what was found for root AMT (Figs. 2C and S1A). In contrast, we find that in this setup, where Cas9 expression does not rely on T-DNA integration, that TMEJ inactivation does not lead to a dramatic reduction in the fraction of mutant alleles (Fig. 2B), arguing that the reduced number of footprints observed in root AMT experiments (Fig. 1B) can be completely attributed to a reduced number of DSBs because of reduced Cas9 expression. Another intriguing observation is an increased fraction of mutant alleles in *ku70* mutant plants (up to 80% of targeting-susceptible alleles; Fig. 2B), which can be explained by proposing that cNHEJ repairs CRISPR-induced breaks not only in an error-prone but also in an error-free manner.

### T-DNA capture in CRISPR-induced DSBs

Upon close inspection of the types of mutations that are typical for TMEJ, we noticed something peculiar. It has previously been reported that Polθ activity occasionally leads to so-called “templated insertions”: deletions that also contain insertions that are templated from sequences flanking the DSB (16). Those templates can be reliably identified for insertions of decent size (≥6 bp). Indeed, when inspecting the delins found in the PPO locus of transformed wild-type roots, for the majority of this category, we found a match in close proximity of the DSB (Fig. S3). However, and to our surprise, we found 4% of inserts to match T-DNA sequences, particularly sequences located at the 5′ and 3′ ends of the T-DNA (Fig. S3C). This observation indicates that at least some DSB ends, prior to being joined to the opposing genomic DSB end, have temporarily interacted with T-DNA, which has served as a template for DNA extension. This apparent interaction also suggests that the same mechanisms facilitating EJ repair, also facilitate T-DNA capture at Cas9-induced breaks.

**Fig. 3.**
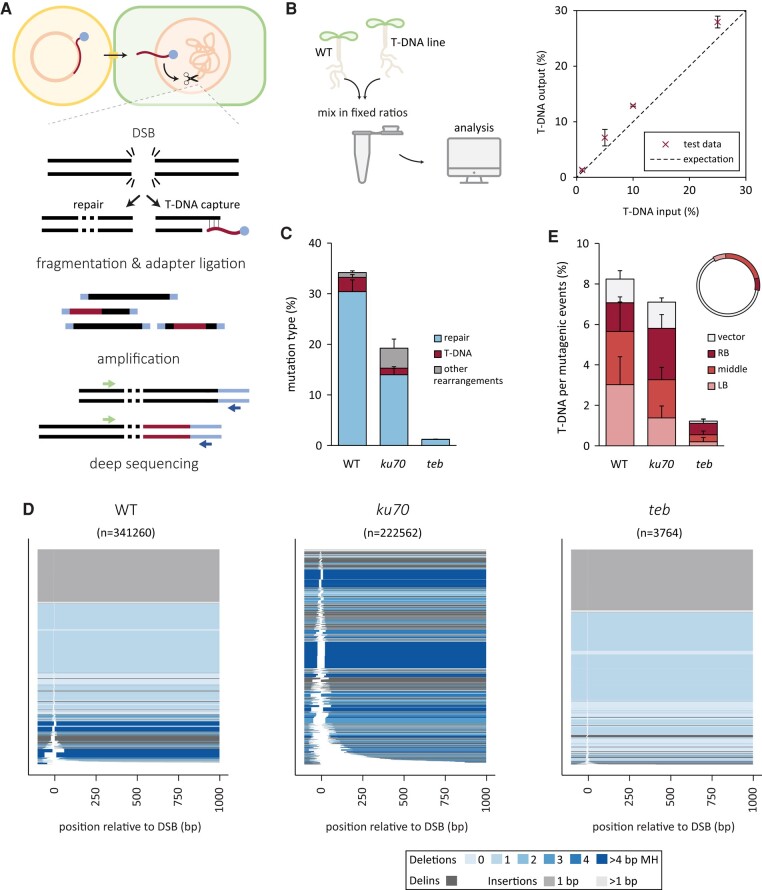
Unbiased identification of all interactants of the Cas9-induced DSB using CISGUIDE method. A) A schematic overview of the experiment. T-DNA is transferred from *A. tumefaciens* to roots of *A. thaliana* seedlings. Cas9 and the sgRNA are expressed from the T-DNA and a DSB is induced in the PPO locus, which can undergo interaction with other DNA ends (derived from genomic DNA or T-DNA). After isolation of the genomic DNA, the DNA is fragmented and adapter sequences are ligated to the ends. Junctions between the DSB flank and its interactant are subsequently enriched by PCR using one primer binding to the flank and one binding to the adapter. The resulting amplicons are deep sequenced. This experiment was performed for both flanks of the induced DSB. B) Verification of CISGUIDE outcomes. Left: schematic overview of the experiment. DNA of seedlings from wild-type and a stable T-DNA line is mixed in fixed ratios and processed. Right: percentages of T-DNA events identified by CISGUIDE compared with the input percentages. C) Percentage of nonwild-type sequencing reads. Repair indicates the distal end of the sequencing read contained the other flank of the break. T-DNA indicates the distal end contained part of the T-DNA. Other rearrangements indicate the distal end contained another sequence. The error bars represent the SE between biological replicates. D) Spectra of mutations of all events categorized as repair for the indicated genotypes combined for all biological replicates and both orientations. The relative position on the *x*-axis includes the expected DSB position at 0 bp. All mutational events are stacked and sorted based on their deletion size. The number of reads representing a specific outcome is represented by the thickness of the respective bar. The events are color-coded based on the type of event and the extent of microhomology (MH). E) Percentage of T-DNA events normalized for the percentage of mutagenic events depicted in B, divided in location on the T-DNA or binary vector backbone. LB and RB indicate a window of 200 bp around the respective border. Middle indicates any other location on the T-DNA. Vector indicates a position on the binary vector outside of the T region.

We next set out to investigate this phenomenon in more detail, and to this end, we developed an NGS-based method that allows the unbiased identification of sequences attached to a given CRISPR-induced break end. We called this method, which is an adaptation to the recently developed TRANSGUIDE workflow (27), CISGUIDE, for CRISPR-induced Sequence Gain/loss, Unbiased Identification (schematically illustrated in Fig. 3A). Genomic DNA is isolated from root calli after transformation with T-DNA-encoding CRISPR reagents. After fragmentation of the DNA, adapter sequences are ligated, and PCR is performed using a locus-specific primer (in close proximity to the sgRNA target) and a primer binding to the adapter. Amplicons are subsequently analyzed by NGS. The outcomes are sequence reads that cover thousands of junctions in which one end of the DNA break (dictated by primer design) is connected to any other sequence, which can be the other break end, T-DNA, or other parts of the genome, the latter of which may reflect translocations or other forms of genome rearrangements. To test this new method, we assayed DNA samples in which wild-type DNA and DNA of a well-studied T-DNA insertion line (SALK_044027) were mixed in fixed ratios. Confirming the credibility of CISGUIDE, we found the ratios of reads containing T-DNA to match the expected values (Fig. 3B).

We next conducted CISGUIDE on DNA isolated from root calli transformed with Cas9, and found 34% of the reads to contain alterations (Fig. 3C), most of which are also identified by amplicon NGS (compare Fig. 1C with Fig. 3D), again confirming the credibility of CISGUIDE. However, being less restrictive to size (only one, instead of two, locus-specific primer needs to be retained) CISGUIDE additionally identifies larger deletions, which are especially manifest in *ku70* mutant plants, hence representing TMEJ action (Fig. 3D). Consistent with previous data (Fig. 1B), we found a greatly reduced number of reads containing alterations in *teb*, due to a reduced Cas9 expression caused by the mutant's resistance to T-DNA integration.

Apart from joined DSB ends, CISGUIDE identified 3% of mutagenic reads in which one DSB end is connected to sequences that map to other parts of the *Arabidopsis* genome, reflecting genome rearrangements. Importantly, 8% of mutagenic reads are joined segments of the inflicted DSB site and the T-DNA sequence, indicative of T-DNA capture. Notably, these capture events are not exclusively resulting from joining the 5′ or 3′ end of T-DNA to the CRISPR-induced break (Fig. 3E), indicating that also other regions of the T-DNA and even vector backbone are interacting with the genomic DSB, in line with the T-DNA shards that were found between deletion junctions (Fig. S3C). In agreement with the previously proposed role for Polθ in attaching the 3′ (LB) end of T-DNA to DSBs in the host genome (19, 27), almost all residual cases in the *teb* mutant mapped to the 5′ (RB) end or to middle segments of T-DNA.

### Mutational footprints in T-DNA capture

**Fig. 4.**
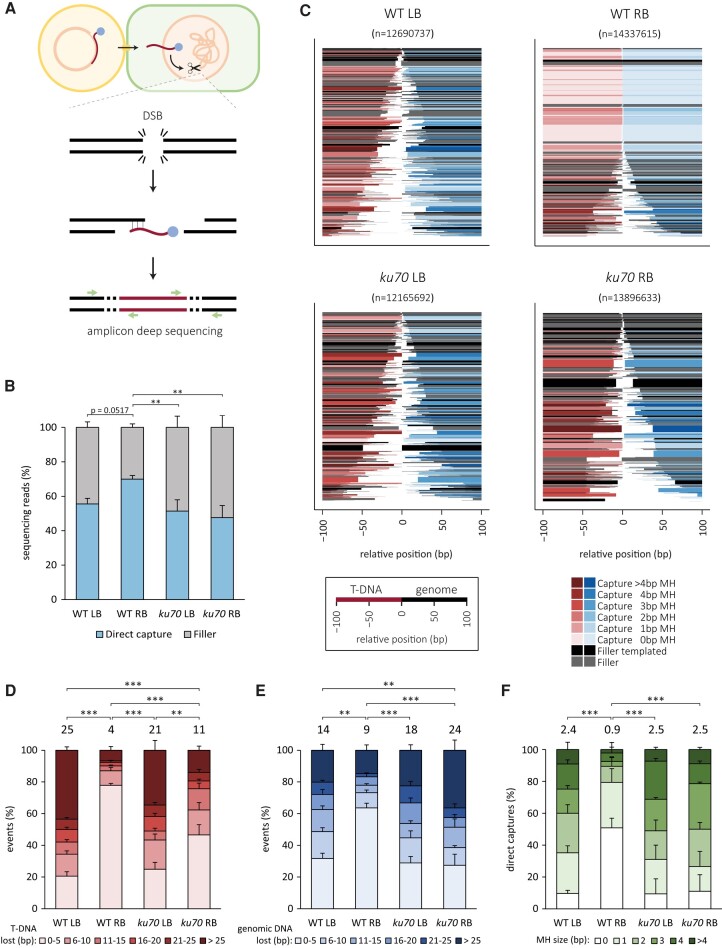
Mutational footprints in junctions between the genome and the T-DNA LB or RB. A) A schematic overview of the experiment. T-DNA is transferred from *A. tumefaciens* to roots of *A. thaliana* seedlings. Cas9 is expressed from the T-DNA and a DSB is induced, which can result in capture of the T-DNA. After isolation of the genomic DNA, the junction between the genome and respectively LB or RB is amplified and deep sequenced. B) Percentage of sequencing reads with or without filler sequence. The error bars represent the SE between biological replicates. Statistical significance was calculated by Kruskal–Wallis test, followed by a post hoc Wilcoxon rank-sum test with Bonferroni correction for multiple testing. **P ≤ 0.01. C) Spectra of mutations occurring in the T-DNA–genome junction for the indicated genotypes and borders, combined for all biological replicates. All mutational events are stacked and sorted based on their size. The number of reads representing a specific outcome is represented by the thickness of the respective bar. The events are color-coded based on the type of event and the extent of microhomology (MH). The relative position on the *x*-axis includes the expected T-DNA end and DSB position at 0 bp. Negative values indicate the T-DNA flank (red/gray) and positive values indicate the genomic flank (blue/gray). D) Histogram depicting the deletion size on the T-DNA flank of all direct capture events. E) Histogram depicting the deletion size on the genomic flank of all direct capture events. F) Histogram depicting the microhomology use of all direct capture events. D–F) The error bars represent the SE between biological replicates. The weighted average is indicated on top of the bars. Statistical significance between the weighted averages was calculated by Kruskal–Wallis test, followed by a post hoc Wilcoxon rank-sum test with Bonferroni correction for multiple testing. **P ≤ 0.01, ***P ≤ 0.001.

Having established a high frequency of T-DNA–genome junctions at CRISPR-induced DSBs, which serves as a proxy for T-DNA capture, we next wished to identify the underlying molecular mechanisms and study the molecular parameters at high resolution. To this end, we employed a directed NGS approach (schematically illustrated in Fig. 4A) to obtain hundreds of genome–T-DNA junctions upon root transformation of wild-type and *ku70* mutant plants (Fig. 4); transformation of *teb* mutant plants was not included in this analysis because of the defect in T-DNA integration. We further restricted our analysis to genomic capture of either the T-DNA LB or the RB, which make up the majority of events (Fig. 3D). As for genomic targets, we analyzed both DSB ends of three different loci (i.e. *PPO*, *CRU3*, and *ADH1*), which thus provide six datasets per T-DNA border. Figure 4 represents the data in which we have compiled all loci together (separate plots are visualized in Fig. S4).

Separating capture events into two categories, i.e. those containing and those being devoid of “filler DNA”/templated insertions (Fig. 4B), reveals a distinction in the biochemistry of LB and RB attachments to CRISPR-induced breaks: LB attachment more frequently goes together with filler DNA at the junction, which is a hallmark of TMEJ action. A reduced percentage of filler-containing junctions for RB-genome connections may thus suggest capture via a TMEJ-independent manner. In line with this notion, the features of genome–T-DNA junctions without fillers are also different for LB and RB connections (Fig. 4C–F). The LB-genome junctional profile is very typical for TMEJ: prevalent microhomology at the junctions (2.4 bp on average; Fig. 4F) and loss of sequence either at the T-DNA or genome side (Fig. 4D and E). These features are also observed for roughly 50% of RB-genome junctions (Fig. 4C), but another predominant class is observed, i.e. junctions not characterized by microhomology and having not lost any (19%) or only a few bp of DNA. These characteristics are suggestive of direct ligation of blunt ends. The analysis of T-DNA capture in *ku70* mutants argues that it is cNHEJ which is responsible for attaching up to 50% of RB T-DNA ends to CRISPR breaks. In this mutant background the features for LB and RB attachment are very similar if not identical: no statistical difference for the fraction of junctions containing fillers (Fig. 4B) nor for microhomology found at the junctions of connections devoid of fillers (Fig. 4F).

**Fig. 5.**
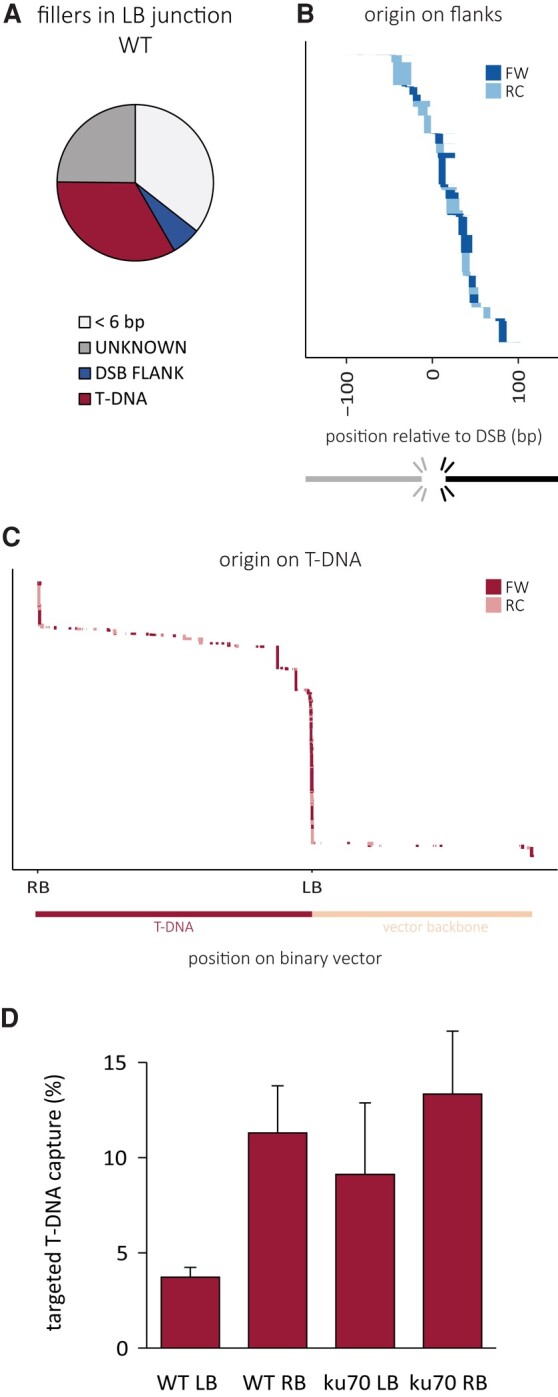
Origin of fillers in LB-genome junctions in wild-type. A) Distribution of fillers. Fillers smaller than 6 bp were not mapped. Fillers that were reliably mapped to either of the flanks or the T-DNA vector sequence are indicated. Unknown fillers could not be reliably mapped. B) Fillers mapping to the genomic flank. Negative values indicate the DSB flank not participating in the T-DNA junction. Positive values indicate the flank present in the junction. C) Fillers mapping to the T-DNA vector. All templated filler events are stacked and sorted based on their position on the reference sequence. Negative values on the *x*-axis indicate the T-DNA flank up to the expected nick site. Positive values indicate position on the binary vector downstream of the expected nick site. The number of sequencing reads representing a specific outcome is represented by the thickness of the respective bar. The size of the templated insertion is represented by the width of the bar. The events are color-coded based on their orientation relative to the reference: FW, forward; RC, reverse complement. D) Targeted vs. random T-DNA integration. T-DNA junctions that are within a 1,000-bp region around the induced DSB site are considered targeted. Targeted events are presented as a percentage of all recovered T-DNA junctions for the indicated genotypes and borders.

Together, these data indicate that T-DNA is captured at CRISPR-induced DNA breaks at an unexpectedly high frequency (8%) and that capture of the 3′ LB requires TMEJ, while the 5′ RB can be joined via TMEJ as well as cNHEJ. We find that the genomic break ends and T-DNA ends compete with each other, as in scars of DSB repair small shards of T-DNA are found (Fig. S3), and in T-DNA genome attachments, small segments (“templated insertions”) are found that either map to the opposing CRISPR break end (Fig. 5B) or map to the other end of the T-DNA (Fig. 5C), which thus has temporarily served as an intermediate template for Polθ action.

### Targeted vs. random T-DNA integration

**Fig. 6.**
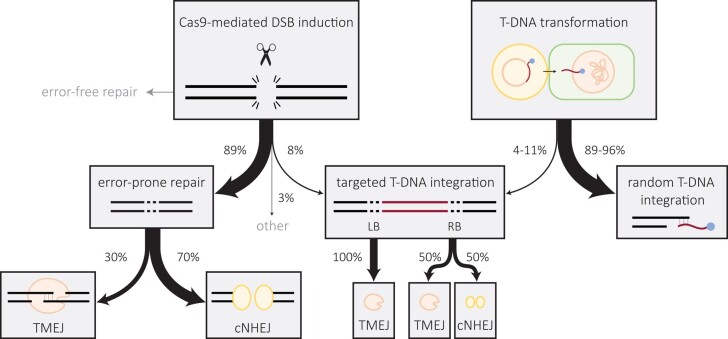
Scheme integrating all outcomes of our studies, which highlights the interrelation between repair of Cas9-induced breaks and T-DNA integration, and the pathways mediating them. The frequencies shown are obtained within this study.

Having determined the frequency with which T-DNA is captured at CRISPR-induced DSBs, we next wished to compare this to the frequency of T-DNA integration at random genomic positions. To this end, we used an adapted version of TRANSGUIDE, which enables the identification of genomic DNA sequences that are attached to T-DNA upon integration (27). Using this methodology, we found that in wild-type, ∼4% of all detected T-DNA LB capture events are within the targeted locus (Fig. 5D). For the RB, we found ∼11% of capture events to map to the locus. Together, our data demonstrate that similar mechanisms govern DSB repair and T-DNA integration and that exogenously provided DNA (T-DNA) can substitute for genomic DSB ends during repair of chromosomal breaks. Our quantitative analysis provides strong support for the notion that CRISPR-induced DSBs can serve as a remarkably proficient entry site for T-DNA integration (Fig. 6).

## Discussion

The recent emergence of CRISPR technology has boosted the possibilities for precise genome engineering in plants: a great body of research performed over the past few years has firmly established that for many plant species targeted mutagenesis can be brought about via mutagenic EJ of CRISPR-induced DSB repair (38–46). In a parallel development in that same period, it has become increasingly clear that mutagenic EJ in both plant and mammalian species is not exclusively resulting from the action of the well-studied cNHEJ pathway (47–51). In fact, for multiple species, it has now been demonstrated that genomic scars induced by (primarily replication-associated) DSBs are the result of Polθ action, in a pathway nowadays frequently referred to as TMEJ (52–54).

Here, we demonstrate that the EJ pathways TMEJ and cNHEJ together are responsible for the vast majority of CRISPR/Cas9-induced mutation induction in the model plant *A. thaliana*. We used NGS-based technology to establish mutation profiles in genetic mutants that are defective specifically in one of both pathways, and as such are able to define pathway-specific features for mutagenic repair of CRISPR/Cas9-induced DSBs in plants. Furthermore, we reveal an unexpectedly high level of capture of *A. tumefaciens* T-DNA within CRISPR/Cas9 DSBs, and define the genetic requirements for this process, which are dissimilar for the different ends of T-DNA: capture of the 3′ end at CRISPR/Cas9 DSB ends is the exclusive action of TMEJ, while capture of the 5′ end can be accomplished by both TMEJ as cNHEJ. Finally, we found evidence for plentiful interactions between recombinogenic DNA ends prior to the completion of EJ, manifesting as T-DNA shards within genomic deletions, which argues for much more primer-template dynamics during TMEJ than was previously recognized.

### cNHEJ and TMEJ produce different types of mutations at CRISPR/Cas9 DSBs

It is becoming increasingly clear that TMEJ, which is also referred to as alternative or backup EJ, not only operates as an alternative/backup mechanism to cNHEJ, but can also act in cNHEJ proficient conditions, as we also demonstrate here for repair of CRISPR-induced DSBs in *Arabidopsis* root and germline tissues. Based on the pathway-defining mutational signatures derived in mutant cells, we estimate that in wild-type *Arabidopsis* ∼30% of CRISPR-induced mutations are contributed by TMEJ and the other 70% by cNHEJ. These percentages are not reflecting the actual contribution of both pathways to DSB repair in general: while TMEJ is intrinsically mutagenic, cNHEJ can also repair breaks with high fidelity (8, 55, 56), as we have also observed in this study for plants that constitutively express Cas9. In these plants, we found cNHEJ deficiency to reduce the number of wild-type alleles, which argues that in cNHEJ proficient conditions, these alleles had acquired a DSB but were repaired accurately. Similar to the mutational profile established in other genetic systems (13, 15, 56), we found in *Arabidopsis* that mutagenic cNHEJ is *grosso modo* independent of the sequence context surrounding a DSB, and predominantly produces small deletions with little use of microhomology, and 1 bp insertions. The latter are a typical manifestation of cNHEJ repair at CRISPR/Cas9-induced breaks (35, 57, 58) yet not found equally frequent at different target sites. As these 1 bp insertions are considered to result from fill-in synthesis and EJ of DSBs having a 1-nt protrusion, it may be that the tendency of Cas9 to create staggered, as opposed to blunt, DSBs is sequence context dependent.

In contrast to cNHEJ, TMEJ scars are dependent on the sequence surrounding the DSBs (15, 34), because Polθ uses microhomologous sequences (our data suggest that in *Arabidopsis* microhomology of on average 3 bp is used) in the DSB-flanking region to prime DNA synthesis. As a result of this necessity, TMEJ deletions are larger than those produced by cNHEJ. However, microhomology availability is likely not the only reason for this difference, as two observations hint toward processing of DSB ends prior to TMEJ action: (i) in our CISGUIDE experiments, we found a substantial fraction of TMEJ deletions that are much larger, in fact, running into a kb-size range. It is presently unknown whether this reflects loss of DNA at the 3′ end, or, alternatively, whether these larger deletions are the products of newly formed 3′ molecules, for instance when a replication fork approaches a DSB that experienced 5′ to 3′ end resection, potentially creating a more distant 3′ end. Recent studies in mammalian cells point to TMEJ acting just prior to or during mitosis, which supports the idea that DSBs that are induced in pre-replicative DNA can be converted into two broken sister chromatids by forks converging from either end of the DSB (59); (ii) junctional analysis of T-DNA capture events reveals that in *ku70* mutants, in which all capture is TMEJ-mediated, more T-DNA is retained at the RB end than at the LB end (Fig. 4D). This phenomenon, which is also evident in data describing random integration of T-DNA (60, 61) may be because the RB, but not the LB, is protected from exonucleolytic attack during transport toward the plant nucleus by the covalently bound VirD2 protein.

### T-DNA shards in Cas9-induced DSBs

A typical signature hallmark for TMEJ action, which is also clearly visible in the CRISPR-induced mutation profiles in *Arabidopsis*, is the presence of templated insertions in between the junctions of a deletion (16) and references therein. Current models explain these Polθ footprints by iterative rounds of microhomology-mediated DNA extension followed by release of the extended end from the template. These insertions, also called “fillers” in plants, can thus be considered telltales of previous temporary interactions between two molecules: one that served as a primer and the other as a template. Oftentimes, these insertions map to the immediate flank of the deletion and, hence, can be interpreted as local primer-template switching (17, 62). However, in our analysis of CRISPR-induced breaks, we surprisingly found insertions in between the deletion junction that originate from T-DNA sequences, in particular, the 5′ and 3′ ends. The presence of such T-DNA shards can be explained by proposing that prior to joining of the genomic DSB ends, one end interacted with (the reactive end of a) T-DNA in a TMEJ reaction, where the genomic end served as a primer and the T-DNA as a template. EJ of this extended end to the opposing end of the genomic DSB results in a deletion having a small segment of T-DNA caught in the middle. Only ∼50% of insertions are of sufficient size to have their origin reliably determined and of those ∼8% map to T-DNA. A number of implications follow from this finding. First of all, CRISPR-induced DSB ends may be much more “open,” i.e. more susceptible to reacting with other DNA molecules as opposed to with each other, than previously assumed. It may thus be, as discussed above, that DSBs requiring TMEJ undergo yet undefined DNA flexibility that separate the reactive genomic ends. Secondly, TMEJ is much more dynamic than previously envisaged: in several mechanistic models for TMEJ, templated insertions are proposed to result from local primer-template switches, perhaps even while the enzyme remains DNA-bound. However, we here find that insertions frequently originate from completely different donor molecules that “happen to be” in the neighborhood. It is thus not a given that templated insertions mapping to the flank of the DSB are resulting from a juxtaposed configuration of DSB ends. Finally, from a genome engineering perspective, a T-DNA molecule could be a viable vehicle to insert desired sequences into the plant genome at CRISPR/Cas9 sites.

### Two mechanisms for attaching T-DNA's 5′ end to CRISPR/Cas9 DSBs

Using the newly developed CISGUIDE methodology, we quantified T-DNA capture at CRISPR-induced DSBs to account for ∼8% of all mutant alleles. The majority of these events consisted of the capture of the T-DNA's 5′ and 3′ ends, in line with these ends being reactive toward EJ machineries. In random integration, the vast majority of T-DNA–genome junctions comprises the T-DNA LB (3′) end (27, 60), supporting a model in which the entry sites for T-DNA integration are replication-associated breaks, which are processed to have 3′ protruding tails. These breaks are the main substrate of TMEJ (53, 63), whereas cNHEJ cannot operate on them as the KU heterodimer only binds dsDNA ends (64, 65).

In contrast to replication-associated DSBs, CRISPR/Cas9-induced DSBs are (at least directly following endonuclease action) predominantly blunt-ended, which principally allows for cNHEJ-mediated capture. Making use of NGS-based methods to directly address T-DNA capture biology at these DSBs, we found a profound contribution of cNHEJ, but exclusive to capture of the RB end, which suggests that a significant fraction of T-DNA's RB ends has a (near) blunt double-stranded configuration prior to its capture. Based on the mutational hallmarks typifying TMEJ in *Arabidopsis* presented in this study, the RB end can also be attached to the genome by this pathway, accounting for ∼50% in wild-type, and for 100% in cNHEJ-deficient plants—these hallmarks are equally widespread in the junctional spectrum of either LB-genome or RB-genome attachment in *ku70* mutants. At present, we have no information to infer a preferred order of events, or potential interdependencies (RB capture only upon LB capture or vice versa) in T-DNA integration into CRISPR-induced DSBs.

Our finding that about 10% of T-DNA integrations are at the targeted locus, but 90% are not, raises questions about the source and number of “random” genomic breaks that are at the origin of the majority of integration events upon AMT. For instance, is there (at least) a 10-fold excess of spontaneously occurring DSBs over CRISPR-induced DSBs in the window in which TMEJ can act? TMEJ was demonstrated to act on replication-associated DNA breaks originating at replication-blocking DNA damage or at thermodynamically stable secondary structures (53, 66), and more recent studies point to G2 and mitosis as cell cycle phases permissive to Polθ action (59, 67). However, whole-genome sequencing of propagated *Arabidopsis* lines has revealed little, if any, mutations fitting a typical TMEJ signature (68), arguing that DSBs that are in need of TMEJ to maintain an intact genome are rare during normal growth. Paradoxically, successfully transformed plant cells frequently have multiple sites where T-DNA copies have integrated, arguing for the presence of numerous DSBs that tolerate TMEJ action. It is thus presently all but clear whether T-DNA integrates at sites of spontaneously occurring DSBs, or whether, e.g. the process of AMT itself is stimulatory to DSB formation. In this light, it is of interest that a recent study demonstrated elevated levels of mutations in yeast cells expressing the *Agrobacterium* protein VirD5, which during AMT in plants is transferred into the host (69).

## Concluding remarks

Genome engineering in plants is becoming an increasingly important research area given the impact of a growing global population and a rapidly changing climate on agriculture: crop species producing higher yields and greater tolerance to abiotic stress are needed. CRISPR technology has now created the possibility for precise genome engineering in almost any crop genome to generate novel variation and thereby accelerate breeding strategies. It is thus important to establish the molecular parameters as well as the genetic components that modulate the outcome of CRISPR-induced genome engineering, such that tailor-made approaches can be developed. In addition, detailed insight into how ectopically provided DNA can interact with the genome, in particular with the targeted site, is expected to aid either by preventing undesired integration effects or by stimulating the inclusion of desired stretches of DNA sequence.

## Materials and methods

### Plant lines

All lines used in this study are derived from the Columbia-0 ecotype. T-DNA insertion lines were obtained from the SALK institute T-DNA collection (70). Polθ knockout line *teb-5* (SALK_018851) was described previously (10, 19). cNHEJ mutants *ku70* and *lig4* (SALK_123114 and SALK_044027, respectively) were previously characterized by Jia et al. (71, 72). Double mutants were obtained by crossing the aforementioned lines.

### *A. tumefaciens* strains

All constructs used in this study are based on pDE-Cas9 (42), containing a T-DNA sequence with the PPT resistance gene *bar*, and *CAS9* and sgRNA expression cassettes. pDE-CasPPO, containing a protospacer specific to the protoporphyrinogen oxidase gene (At4G01690), was constructed and described previously (45). pDE-CasCRU and pDE-CasADH, targeting the cruciferin 3 (At4G28520) and alcohol dehydrogenase 1 (At1G77120) gene, respectively, were constructed by Strunks (73). pDE-CasGL2, targeting the *GLABRA2* gene (At1G79840), was constructed using the pDE-Cas9 vector and cloning methods reported by Fauser et al. (42), in combination with oligos making up the protospacer sequence described by Mao et al. (36) (Table S1). The constructs were introduced in the disarmed hypervirulent *A. tumefaciens* strain AGL1 (74) using electroporation (75).

### Root transformation

Root transformations were performed as described by Vergunst et al. (76). After cocultivation the root explants were transferred to shoot induction medium supplemented with 100 mg/L vancomycin, 100 mg/L timentin, and 30 mg/L PPT. After an incubation period of 21 days, pools of 20 calli were frozen in liquid N_2_ and subsequently disrupted in a TissueLyser (Retch). DNA was isolated from disrupted frozen callus material using phenol/chloroform extraction as described by de Pater et al. (77) or using Promega Wizard Genomic DNA Purification Kit, according to the manufacturer's instructions, with the adaptations described by Vorwerk (78). Because of the small amount of callus tissue, 20% of the recommended volumes were used.

### Cas9-expressor lines

Stable Cas9-expressor lines were obtained by crossing the previously described Cas9 expressing line (36, 37) (♂) with the aforementioned *teb-5* or *ku70* mutant (♀). Hygromycin-resistant F_1_ plants were selected by plating F_1_ seeds on half strength Murashige and Skoog medium containing 100 µg/mL nystatin, 100 µg/mL timentin, and 15 µg/mL hygromycin. These were subsequently selfed for the collection of F_2_ seeds. Candidate F_2_ individuals were selected from populations of F_2_ progeny by screening for (i) homozygosity for the respective wild-type (*TEB^+/+^*, *KU70^+/+^*) or knockout (*teb*^−/−^, *ku70*^−/−^) alleles, (ii) heterozygosity for the ΔG *gl2* allele from the parental Cas9 expressor line (*GL2*^+/ΔG^), and (iii) presence of the *pDD45::CAS9* expression construct, via PCR analysis and Sanger sequencing (Macrogen Europe, Amsterdam, The Netherlands) with the primer combinations listed in Table S1. Pools of 400 randomly selected F_3_ progeny of selfed candidate F_2_ individuals were frozen in liquid N_2_ 10 days after germination and subsequently disrupted in a TissueLyser (Retch). DNA was isolated using phenol/chloroform extraction as described by de Pater et al. (77).

### Amplicon deep sequencing

Twenty-five nanograms of genomic DNA were used as a template for PCR amplification with Phusion polymerase (Thermo Fisher) in a final volume of 25 μL. Primers used for each amplicon are listed in Table S1. Genomic repair junctions were subjected to 25 amplification cycles, whereas T-DNA–genome junctions were subjected to 35 cycles. PCR products were purified using Ampure XP beads (Beckman Coulter). A secondary PCR was performed in a final volume of 20 μL for 5 cycles to add NGS barcodes to the amplified junctions, followed by a second bead purification. DNA concentrations were measured using the Qubit dsDNA HS Assay Kit (Invitrogen). Purified secondary PCR products were pooled in an equimolar ratio. Paired end sequencing was performed using an Illumina NovaSeq6000 (GenomeScan BV, Leiden, The Netherlands). Data analysis was performed using the sequence analyzer described in van Schendel et al. (79).

### Statistical analysis

A weighted average of deletion, insertion, or microhomology size was computed for each replicate, taking into account the number of reads found for each individual event. We then compared the weighted averages for each junction, comprising at least three biological replicates, using the Kruskal–Wallis test, followed by a post hoc Wilcoxon rank-sum test with Bonferroni correction for multiple testing.

### CISGUIDE and TRANSGUIDE

Junction enrichment from purified callus DNA was performed as described by Kralemann et al. (27), using the primers listed in Table S1. Sequencing was performed using an Illumina NovaSeq6000 (GenomeScan BV) and Illumina MiSeq (Leiden Genome Technology Center, Leiden, The Netherlands), and reads were mapped to the *Arabidopsis* TAIR10 genome as described (27). Reads were then annotated using custom-made software that positions the nonprimer flank using the second-in-pair read, with the requirement that the first-in-pair read mapped to the same chromosome. This program differs from the program in Kralemann et al. (27) to accommodate the requirements of the CISGUIDE procedure: this software analyses read pairs individually, and afterwards groups them based on outcome. Events supported by <3 unique read pairs were discarded. For CISGUIDE, the events were classified as: WT (region surrounding the induced DSB site was identical to reference), Repair (nonprimer flank mapped to chromosome 4 in the correct orientation), T-DNA (nonprimer flank mapped to pDE-Cas9-PPO reference sequence); Other (nonprimer flank mapped to other chromosome or chromosome 4 in reverse orientation). Repair events were subsequently divided into Deletion, Delins, or Insertion. To avoid artifacts induced by PCR or sequencing, single nucleotide variations were not taken into account. For TRANSGUIDE, the events were classified as: Targeted (nonprimer flank mapped within 1,000 bp region around the induced DSB site) or Random (nonprimer flank mapped elsewhere in the reference genome).

## Supplementary Material

pgae094_Supplementary_Data

## Data Availability

Data that support the findings of this study are available within the article and its supplementary material, and openly available from NCBI SRA, PRJNA885005.
