# Supplementary material for: Genetic dissection of mutagenic repair and T-DNA capture at CRISPR-induced DNA breaks in Arabidopsis thaliana
Source: PNAS Nexus. 2024 Feb 26;3(3):pgae094. doi: 10.1093/pnasnexus/pgae094 (PMC10923293; doi:10.1093/pnasnexus/pgae094)
Supplement: pgae094_Supplementary_Data [file pgae094_supplementary_data.pdf]

Supporting Information for

Genetic dissection of mutagenic repair and T-DNA capture at CRISPR-induced DNA breaks in *Arabidopsis thaliana*

Lycka Kamoen[1], Lejon E. M. Kralemann[1,2], Robin van Schendel[2], Niels van Tol[1,2], Paul J. J. Hooykaas[1], Sylvia de Pater[1] & Marcel Tijsterman[1,2].

[1]Department of Plant Sciences, Institute of Biology Leiden, Leiden University, Sylviusweg 72, 2333 BE, Leiden, The Netherlands

[2]Department of Human Genetics, Leiden University Medical Center, Einthovenweg 20, 2300 RC, Leiden, The Netherlands

*Corresponding author: Marcel Tijsterman

Email: M.Tijsterman@lumc.nl

This PDF file includes:

Figures S1 to S4
Table S1

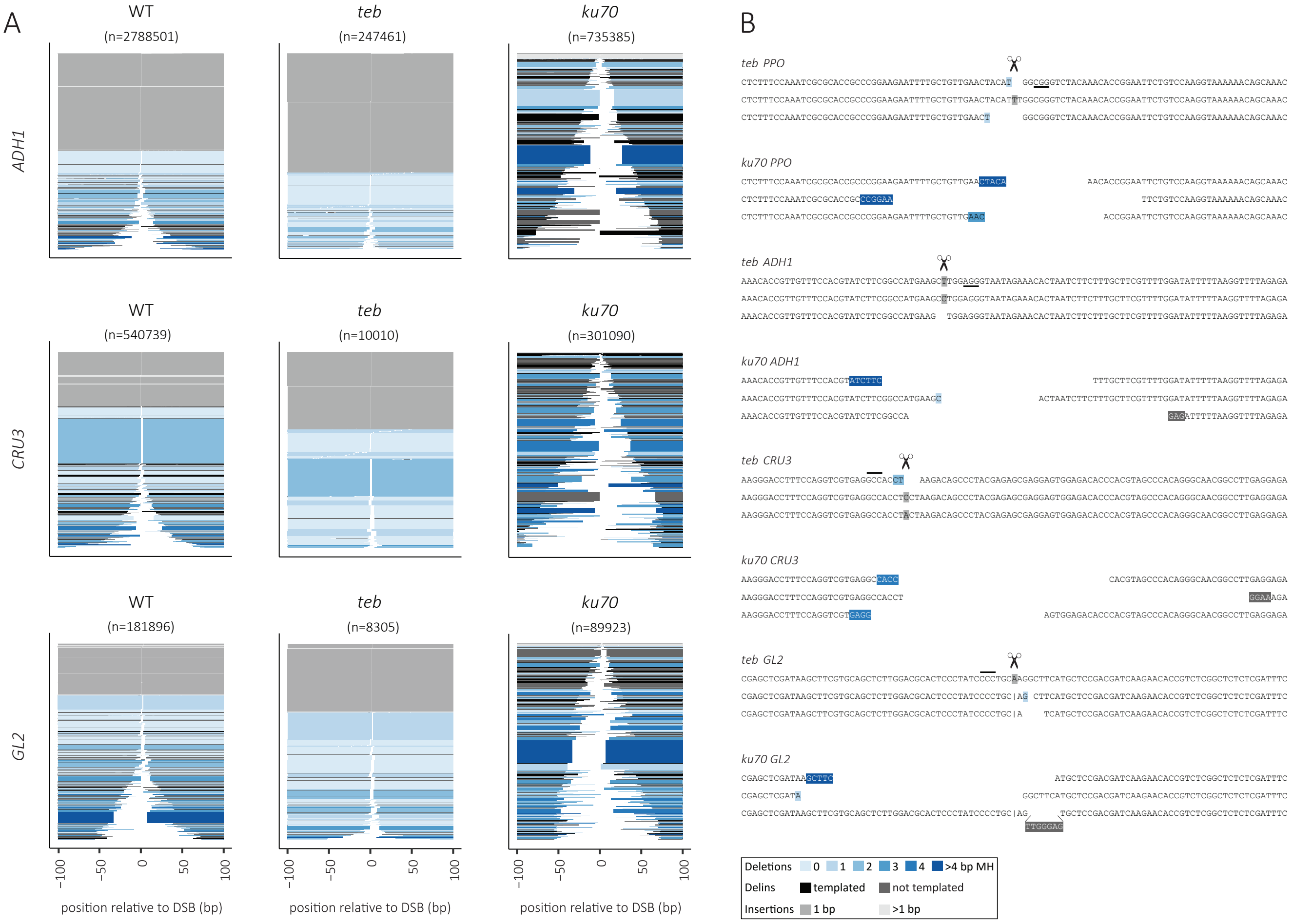

Figure S1. Mutational footprints in the ADH1, CRU3 and GL2 loci. (A) Spectra of mutations occurring in the respective loci and genotypes combined for all biological replicates. The relative position on the x-axis includes the expected DSB position at 0 bp. All mutational events are stacked and sorted based on their size. The number of sequencing reads representing a specific outcome is represented by the thickness of the respective bar. The events are color-coded based on the type of event. (B) Three most preferential outcomes for PPO, ADH1, CRU3 and GL2 loci in *teb* and *ku70* mutants. Expected cut sites are indicated with scissors and the protospacer adjacent motifs are underlined (NGG) or overlined (CCN). Microhomology at the junctions is indicated in blue. Insertions are indicated in grey.

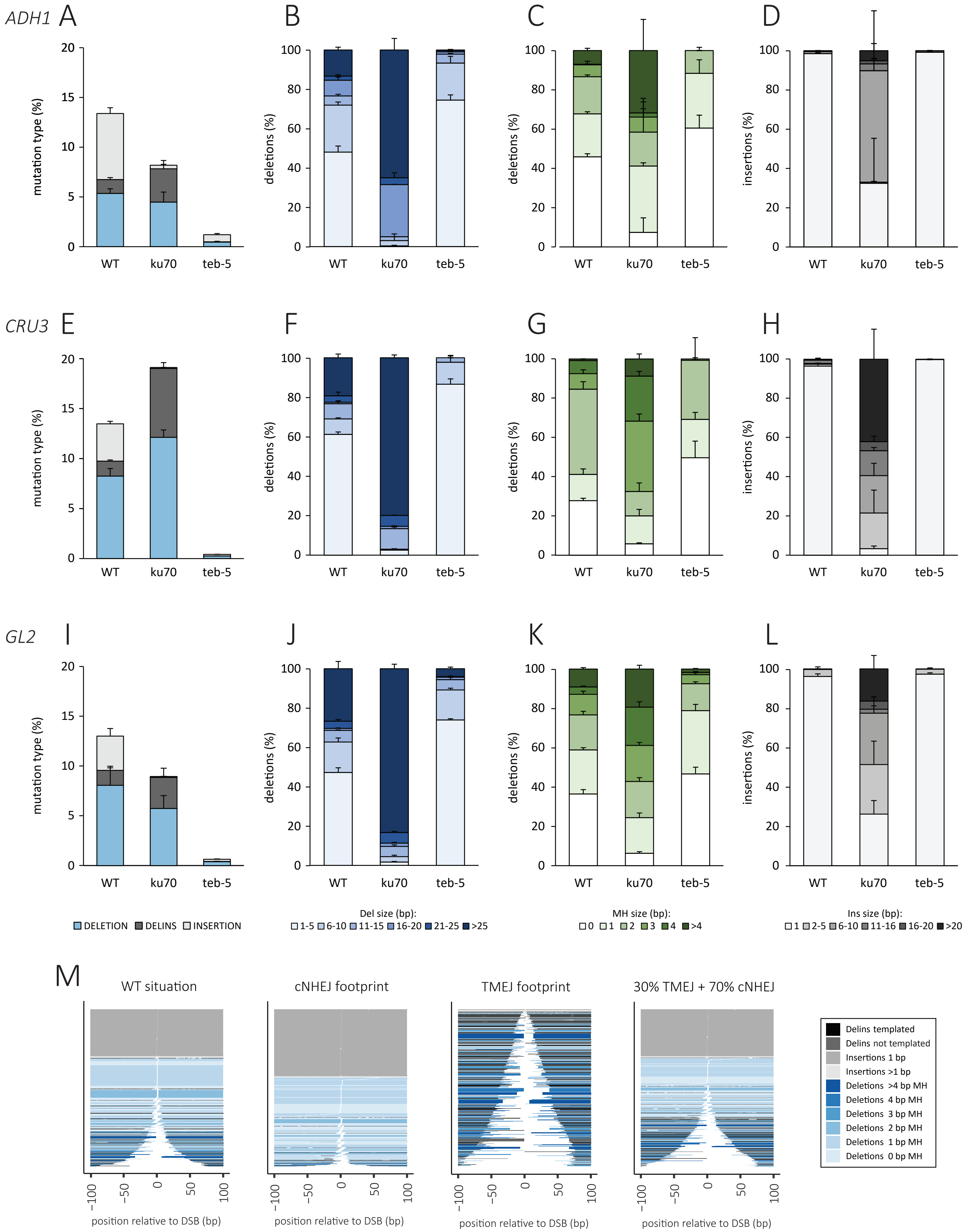

Figure S2. Mutational footprints in the ADH1, CRU3 and GL2 locus. The error bars represent the standard error between biological replicates. (A;E;I) Percentage and type of mutations. (B;F;J) Histogram depicting the deletion size of all deletion events. (C;G;K) Histogram depicting microhomology at the junction of deletion events. (D;H;L) Histogram depicting the insertion size of all insertion events. (M) Combined mutational footprint of all four studied loci. The left panel represents the wild-type situation, the middle panels represent the situation in mutant proficient in only one end joining pathway. The right panel represents a combination of TMEJ and cNHEJ footprints in a 3:7 ratio, which accurately fits the spectrum obtained in wild-type.

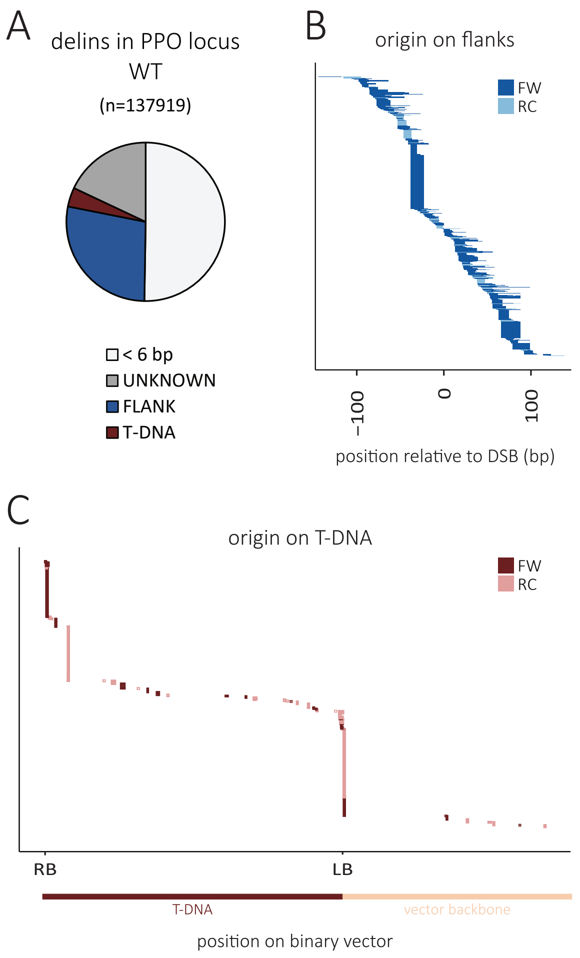

Figure S3. Origin of delins in DSB repair products of the PPO locus in wild-type combined for all biological replicates. (A) Distribution of delins. Delins smaller than 6bp were not mapped. Delins that were reliably mapped to either of the flanks, or the T-DNA vector sequence are indicated. Unknown fillers could not be reliably mapped. (B) Templated insertions mapping to the flanks of the DSB. (C) Templated insertions mapping to the T-DNA vector. All templated insertion events are stacked and sorted based on their position on the reference sequence. The number of sequencing reads representing a specific outcome is represented by the thickness of the respective bar. The size of the templated insertion is represented by the width of the bar. The events are color-coded based on their orientation relative to the reference.

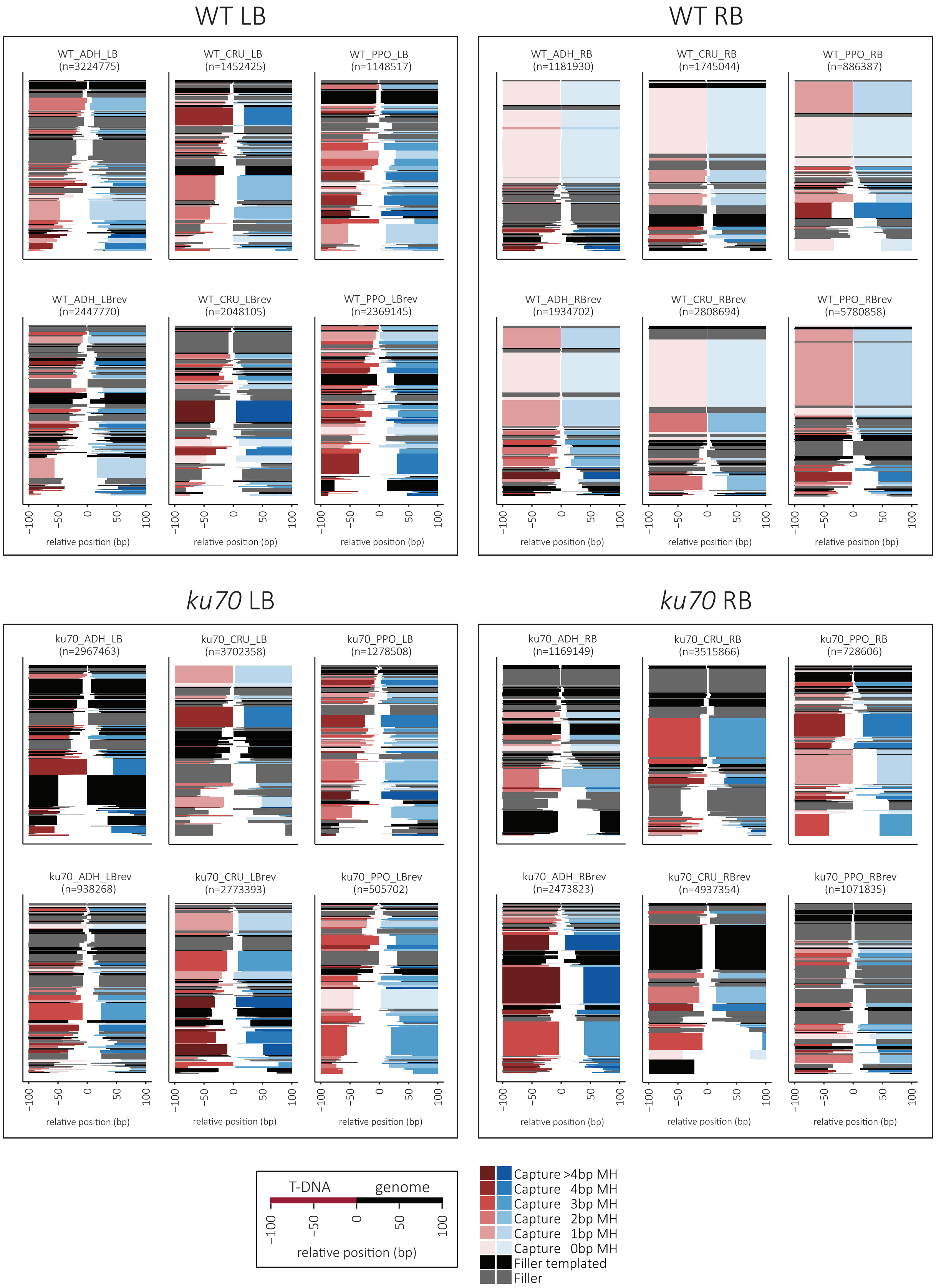

Figure S4. Mutational footprints in junctions between the genome and the T-DNA LB or RB. Spectra of mutations occurring in the T-DNA-genome junction for the indicated genotypes and borders. Each plot represents all biological replicates for one individual junction type (locus and orientation). The relative position on the x-axis includes the expected DSB position at 0 bp. All mutational events are stacked and sorted based on their size. The number of sequencing reads representing a specific outcome is represented by the thickness of the respective bar. The events are color-coded based on the type of event. The relative position on the x-axis includes the expected T-DNA end and DSB position at 0 bp. Negative values indicate the T-DNA flank (red/grey) and positive values indicate the genomic flank (blue/grey).

Table S1. Primers and oligos used in this study. [XXXXXXXX] represents a 8bp barcode and * represents a phosphorothioate bond.

Primer name	Function	Sequence (5' → 3')	Reference
**Construction of pDE-Cas9-GL2**			
LK075	protospacer GL2 forward	ATTGGTCGGAGCATGAAGCCTGCA	This study
LK076	protospacer GL2 reverse	AAACTGCAGGCTTCATGCTCCGAC	This study
**Genotyping of Cas9-expressor lines**			
SALK_015581 FW	wild-type TEB allele	GCCTCTCTGGATGACAAGAG	van Tol *et al.*, 2021
SALK_015581 RV	wild-type TEB allele	CCTTACTACACTTCCTCAAGC	van Tol *et al.*, 2021
SP119	wild-type KU70 allele	TGGGTTGCACAAGCACTACTGC	Jia *et al.*, 2012
SP120	wild-type KU70 allele	GAATAGCCGGACGGAGTAAAGC	Jia *et al.*, 2012
pBIN LB RV	SALK T-DNA insertion alleles	GCCGTCGTTTTACAACGTCG	van Tol *et al.*, 2021
AtGl2-R	GL2 genotype of F2 individuals	AGTTATAGTAGCTGGTAACAG	Mao *et al.*, 2016
DD45 FW	pDD45::hpCAS9 expression construct	CCGTCAATCCTTTCCCATTCC	This study
hpCAS9 RV	pDD45::hpCAS9 expression construct	GTTGGTGCCGATGTCCAGG	This study
**Junction amplification for amplicon deep sequencing**			
SP655	PPO forward	GATGTGTATAAGAGACAGGCTGTTGCTCGTATTTCTTC	This study
SP656	PPO reverse	CGTGTGCTCTTCCGATCTCAGTTCTTAGGTTTACTTGG	This study
SP671	ADH forward	GATGTGTATAAGAGACAGACTCCAAATTCATAATCAAGTTCT	This study
SP672	ADH reverse	CGTGTGCTCTTCCGATCTCTCTCAACAATCCTACACAACAAC	This study
SP673	CRU forward	GATGTGTATAAGAGACAGCAATGTTCTCGTGGGACCTC	This study
SP674	CRU reverse	CGTGTGCTCTTCCGATCTAAACTTGTGGAGCGGGTTCG	This study
LK081	GL2 forward	GATGTGTATAAGAGACAGTCCACCTCGAAAACTCC	This study
LK082	GL2 reverse	CGTGTGCTCTTCCGATCTGAAATCTCGGCAATACGG	This study
SP667	LB forward	GATGTGTATAAGAGACAGGAGAGGCGGTTTGCGTATTG	This study
LK015	LB reverse	CGTGTGCTCTTCCGATCTGAGAGGCGGTTTGCGTATTG	This study
LK016	RB forward	GATGTGTATAAGAGACAGCTGAATGGCGAATGAGCTTG	This study
SP668	RB reverse	CGTGTGCTCTTCCGATCTCTGAATGGCGAATGAGCTTG	This study
**CISGUIDE & TRANSGUIDE**			
LZ351	CISGUIDE LIG4 primary	AGTCATGAACGTTGCCATATGCAAATAA	This study
LZ352	CISGUIDE LIG4 secondary	TCAGACGTGTGCTCTTCCGATCTGATCTTTTATCAAAACATTTATAGC	This study

LK166	CISGUIDE PPO forward primary	GTTATAATCGTCAATTATGTCAAAATGTTCATAGAATCTTCATGCTG	This study
LK167	CISGUIDE PPO forward secondary	TCAGACGTGTGCTCTTCCGATCTTGCTCGTATTTCTTCAGG	This study
LK168	CISGUIDE PPO reverse primary	GCGGAATCAAAACAAAAGAGAGAGAGAGAAGAAAAC	This study
LK169	CISGUIDE PPO reverse secondary	TCAGACGTGTGCTCTTCCGATCTCAGTTCTTAGGTTTACTTGG	This study
LZ054	TRANSGUIDE pCAS LB primary	AGGCGGTTTGCGTATTGGCTA	Kralemann *et al.*, 2022
LZ056	TRANSGUIDE pCAS LB secondary	TCAGACGTGTGCTCTTCCGATCTCGGCGTTAATTCAGTACATT	Kralemann *et al.*, 2022
LZ058	TRANSGUIDE pCAS RB primary	GCGCAGCCTGAATGGCGAAT	Kralemann *et al.*, 2022
LZ060	TRANSGUIDE pCAS RB secondary	TCAGACGTGTGCTCTTCCGATCTATGAGCTTGAGCTTGGAT	Kralemann *et al.*, 2022
P5_1	P5 primary	AATGATACGGCGACCACCGAGATCTA	Kralemann *et al.*, 2022
P5_2	P5 secondary	AATGATACGGCGACCACCGAGATCTACAC	Kralemann *et al.*, 2022
**Barcoding and deep sequencing**			
P5_# Y adapter	P5 Y adapter part 1 (generic)	AATGATACGGCGACCACCGAGATCTACAC[XXXXXXXX] NNWNNWNNACACTCTTTCCCTACACGACGCTCTTCCGATC*T	Illumina
MiSeq common adapter	P5 Y adapter part 2	[Phos]GATCGGAAGAGC*C*A	Illumina
P5_#	P5 adapter primer (generic)	AATGATACGGCGACCACCGAGATCTACAC[XXXXXXXX] ACACTCTTTCCCTACACGACGCTCTTCCGATC*T	Illumina
P7_#	P7 adapter primer (generic)	CAAGCAGAAGACGGCATACGAGAT[XXXXXXXX] GTGACTGGAGTTCAGACGTGTGCTCTTCCGATC*T	Illumina
